# Do singles or couples live healthier lifestyles? Trends in Queensland between 2005-2014

**DOI:** 10.1371/journal.pone.0192584

**Published:** 2018-02-28

**Authors:** Stephanie Schoeppe, Corneel Vandelanotte, Amanda L. Rebar, Melanie Hayman, Mitch J. Duncan, Stephanie J. Alley

**Affiliations:** 1 Physical Activity Research Group, School of Health, Medical and Applied Sciences, Central Queensland University, Rockhampton, Queensland, Australia; 2 Priority Research Centre for Physical Activity and Nutrition, School of Medicine & Public Health, The University of Newcastle, Callaghan, New South Wales, Australia; Hitit University, Faculty of Medicine, TURKEY

## Abstract

**Objectives:**

To compare the frequency of and trends in healthy lifestyle factors between singles and couples.

**Methods:**

Cross-sectional data from annual surveys conducted from 2005–2014 were used. The pooled sample included 15,001 Australian adults (mean age: 52.9 years, 50% male, 74% couples) who participated in the annual Queensland Social Survey via computer-assisted telephone interviews. Relationship status was dichotomised into single and couple. Binary logistic regression was used to assess associations between relationship status, and the frequency of and trends in healthy lifestyle factors.

**Results:**

Compared to singles, couples were significantly more likely to be a non-smoker (OR = 1.82), and meet recommendations for limited fast food (OR = 1.12), alcohol consumption (OR = 1.27) and fruit and vegetable intake (OR = 1.24). Fruit and vegetable intake was not significantly associated with relationship status after adjusting for the other healthy lifestyle factors. Conversely, couples were significantly less likely to be within a normal weight range (OR = 0.81). In both singles and couples, the trend data revealed significant declines in the rates of normal weight (singles: OR = 0.97, couples: OR = 0.97) and viewing TV for less than 14 hours per week (singles: OR = 0.85, couples: OR = 0.84), whilst non-smoking rates significantly increased (singles: OR = 1.12, couples: OR = 1.03). The BMI trend was no longer significant when adjusting for health behaviours. Further, in couples, rates of meeting recommendations for physical activity and fruit/vegetable consumption significantly decreased (OR = 0.97 and OR = 0.95, respectively), as did rates of eating no fast food (OR = 0.96). These trends were not significant when adjusting for the other healthy lifestyle factors. In singles, rates of meeting alcohol recommendations significantly increased (OR = 1.08).

**Conclusions:**

Health behaviour interventions are needed in both singles and couples, but relationship status needs to be considered in interventions targeting alcohol, fast food, smoking and BMI. Further research is needed to understand why health behaviours differ by relationship status in order to further improve interventions.

## Introduction

Non-communicable diseases (NCDs) such as cardiovascular disease, type 2 diabetes and cancer are highly prevalent and a leading cause of death worldwide [1]. In 2012, 38 million (68%) of the world’s 56 million deaths were attributed to NCDs [1]. Lifestyle factors such as poor diet, physical inactivity, alcohol, smoking, sedentary behaviour, and overweight/obesity are among the top 10 risk factors for NCD-related morbidity and mortality [2]. To reduce the burden of NCDs at the population level, the World Health Organization recommends that adults engage in at least 150 minutes of moderate-intensity physical activity throughout the week, consume five servings of fruits and vegetables a day, quit smoking, limit the amount of alcohol intake, reduce sedentary time, and maintain a normal body weight [3–6].

Despite the importance of living a healthy lifestyle, large proportions of the adult population do not meet these health recommendations [2]. People’s motivation to adopt a healthy lifestyle is influenced by a complex interplay of environmental, economic, social and individual factors [7]. At the individual level, socio-demographics such as sex, age, education and income are well-known determinants of healthy lifestyle behaviours [7,8]. In contrast, the influence of relationship status on healthy lifestyle factors is much less recognised. Existing studies in this area [9–12] have shown that being in a committed relationship is associated with health promoting behaviours. For example, couples tend to have a healthier diet, smoke less and consume less alcohol compared to singles [10,11,13]. However, marriage and cohabiting also carry the potential for encouraging unhealthy behaviours, as couples often perform behaviours like eating, watching TV and drinking alcohol together [14–16]. Further, evidence on the influence of relationship status on physical activity and body weight has been inconsistent [17]. Some studies [18–20] have shown higher rates of physical inactivity and overweight in couples, while other studies [8,21,22] have reported higher rates in singles, or no association with relationship status [23–25].

In developed countries, between 20–40% of the adult population are single [26,27], and people more frequently transition between single and couple status during their lifetime [26]. It is recognised that relationship status influences people’s day-to-day health behaviours [22,23]; however, this area of research is still insufficiently explored. First, most previous studies [8,20,23,28–30] examining the influence of relationship status on healthy lifestyle factors have focused solely on one or two lifestyle factors; few studies [21,22,31] have explored multiple healthy lifestyle factors. This is important as people with a high risk of developing NCDs often practice multiple unhealthy behaviours [1]. Second, many previous studies, especially those relating to physical activity and body weight [32], have shown inconsistent results, and this may be attributed to the use of minority populations, small sample sizes and focusing on one gender only. Third, most studies examining the frequency of healthy lifestyle factors by relationship status have reported results for one time point [8,22,28,32]; few studies [18,33] have investigated how the frequency of healthy lifestyle factors in couples and singles change over time.

This study aimed to investigate the frequency of multiple healthy lifestyle factors in Queensland singles versus couples, and to identify trends in their healthy lifestyle factors between 2005–2014.

## Methods

### Sample

The present study utilised data from a series of independent cross-sectional surveys conducted annually between 2005–2014. The pooled sample included 15,001 Australian adults aged 18 and older who participated in the annual Queensland Social Survey conducted by the Population Research Laboratory at Central Queensland University. The samples sizes in the annual surveys ranged from 1,208–2,537 (2005: n = 1,208, 2006: n = 1,241, 2007: n = 1,212, 2008: n = 1,243, 2009: n = 1,292, 2010: n = 1,261, 2011: n = 1,265, 2012: n = 2,519, 2013: n = 2,537, 2014: n = 1,223). The Queensland Social Survey is an omnibus survey of households in the state of Queensland and is administered via computer-assisted telephone interview (CATI). Prior to each annual Queensland Social Survey, the Population Research Laboratory at Central Queensland University pilot tested all survey questions in a small group of Australian adults (n = 60) to establish sufficient respondent understanding. The sampling of the surveys were stratified by gender to reflect the gender breakdown of the Queensland population. Detailed descriptions of the Queensland Social Survey can be found elsewhere [34]. For this study, we utilised the data on socio-demographic and healthy lifestyle factors. Participants provided informed consent and each survey including this study was approved by the Human Ethics Committee at Central Queensland University. Annual response rates for survey participation ranged between 32–44% which is comparable to those reported in other telephone surveys [35].

### Measures

#### Predictor variables

Socio-demographic factors were measured including sex, age (in years), number of children under 18 years living at the residence (none, one or more children), indigenous status (Aboriginal/Torres Strait Islander, not Aboriginal/Torres Strait Islander), urbanisation (urban, rural), education (≤ 12 years, > 13 years), year of survey (2005–2014), and employment (employed, not employed). The ‘not employed’ category included participants who were retired and those who engaged in full-time home duties. Relationship status was categorised into single (always single, widowed, divorced) and couple (de-facto, married). Couples in a de-facto relationship included people in a committed romantic relationship, either cohabiting or not, and with or without children.

#### Outcome variables

Physical activity was assessed using the Active Australia Questionnaire which measures the duration and frequency of recreational and transport-related walking, as well as moderate and vigorous intensity physical activity during leisure-time [36]. Total physical activity was calculated by summing the time spent in walking, moderate physical activity and vigorous physical activity (weighted by two) as proposed in the Active Australia Questionnaire scoring guidelines [36]. The Active Australia Questionnaire has demonstrated acceptable reliability (ICC = 0.64) [36] and criterion validity (r = 0.61) when compared to an objective accelerometer measure [37]. In line with the Australian physical activity recommendations [38], which recommend that adults engage in at least 150 minutes of physical activity per week, participants were classified as insufficiently active (<150 minutes/week) or sufficiently active (≥150 minutes/week).

TV viewing time was assessed using one item: ‘In hours and/or minutes, what do you estimate was the total time that you spent sitting and watching television in the last week?’ There are currently no specific screen time or sedentary behaviour guidelines for adults apart from the recommendation to minimise the amount of time spent in prolonged sitting, such as during TV viewing [38]. However, Australian research [39] has shown that >2 hours of TV viewing per day is associated with an increased risk of cardiovascular disease and all-cause mortality. Given the increased health risks from >2 hours of TV viewing per day, weekly TV viewing time was dichotomised into ≤14 and >14 hours per week.

Fruit and vegetable consumption were assessed using two items: ‘How many serves of fruit do you eat on a usual day?’ and ‘How many serves of vegetables do you eat on a usual day?’. Serving sizes were explained to participants. A dichotomous outcome variable was calculated based on whether or not participants were meeting the Australian dietary recommendations [40] to consume ≥2 servings of fruit and ≥5 servings of vegetables per day.

Fast food consumption was assessed using one item: ‘In the last week (the last 7 days), how many times did you eat something from a fast-food restaurant like McDonald’s, Hungry Jacks, KFC, etc? This also includes other fast-food and takeaway such as fish and chips, Chinese food and pizza for example’. The Australian dietary guidelines [40] do not include specific recommendations on fast food consumption but they do recommend to limit its intake. Based on the distribution, participants’ reported frequency of fast food consumption was dichotomised into ‘none and ‘at least once a week’.

Smoking status was assessed using a single item: ‘Are you presently a smoker?’ The response options were ‘yes’ or ‘no’.

Alcohol consumption was assessed using one item: ‘During the past 30 days, on the days when you drank, about how many drinks did you drink on average?’ Alcohol consumption was dichotomised based on whether or not participants were meeting the Australian recommendations [41] of ‘no more than two alcoholic drinks per day’.

Body mass index (BMI) was calculated by dividing participants’ self-reported weight by height squared (kg/m²). Using the Australian classifications for overweight and obesity [42], BMI scores were dichotomised into normal weight (< 25) and overweight or obese (≥ 25).

### Statistical analyses

Binary logistic regression was used to assess associations between relationship status (single, couple) and healthy lifestyle factors. For this, separate binary logistic regression analyses were conducted for investigating the ‘frequency of healthy lifestyle factor across all years’ (i.e., collapsed across all years) and ‘trends in the healthy lifestyle factors between 2005–2014’(i.e., year as continuous predictor). In the binary logistic models examining the ‘frequency of healthy lifestyle factor across all years’, the predictor variable of interest was relationship status (single, couple) and the outcome variables included the healthy lifestyle factors (physical activity, TV viewing time, fruit and vegetable consumption, fast food consumption, smoking status, alcohol consumption and BMI). Variables adjusted for were sex, age, education, employment, urbanisation, Indigenous status, number of children under 18 years living at the residence and survey year. In the binary logistic models examining ‘trends in healthy lifestyle factors between 2005–2014’, the predictor variable of interest was survey year and the outcome variables were the healthy lifestyle factors mentioned above. The trend analyses were stratified for relationship status (single, couple) and adjusted for sex, age, education, employment, urbanisation, Indigenous status and number of children under 18 years living at the residence. All regression analyses were conducted both unadjusted and adjusted for all other healthy lifestyle factors. For all regression analyses, odds ratios (ORs), confidence intervals (CIs) and *p*-values were used as indicators of effect size. Chi-square and independent t-tests were performed to assess differences in the socio-demographic and healthy lifestyle factors between singles and couples. Analyses were performed in IBM SPSS Statistics (version 22.0) with significance levels set at *p* < 0.05.

## Results

Descriptive statistics of the sample are presented in Table 1. The mean age was 52.9 (SD = 15.9) years, 50% were male, 1% Indigenous, 74% couples, 52% had more than13 years of education, 58% were employed, 81% lived in urban areas, and 65% had children under 18 years living at their residence. With regards to healthy lifestyle factors, 69% of adults were meeting Australian alcohol recommendations, 59% were meeting the physical activity recommendations and 12% were meeting fruit and vegetable recommendations. Further, 86% of adults were non-smokers, 57% consumed no fast food in the last week and 42% spent less than 14 hours per week viewing TV. Some of the healthy lifestyle factors differed significantly between singles and couples. Initial chi-square and t-test analyses showed that, compared to singles, more couples were meeting the recommendations for alcohol consumption (65% vs 71%; *p* < 0.001), were non-smokers (80% vs 88%; *p* < 0.001) and spent less than 14 hours per week viewing TV (40% vs 43%; *p* < 0.001). However, fewer couples than singles had a healthy weight (37% vs 42%; *p* < 0.001).

**Table 1:** Descriptive statistics of the sample (N = 15,001).

	All	Singles	Couples	*P*-value
**Socio-demographic factors, %**				
Age				0.189
(mean (SD))	52.9 (15.9)	52.6 (20.0)	53.0 (14.2)	
(median (IQR))	53.0 (24.0)	55.0 (31.0)	53.0 (22.0)	
Sex				<0.001
Male	50.1	45.6	51.7	
Female	49.9	54.4	48.3	
Education				
13+ years	51.8	47.7	53.3	
0–12 years	48.2	52.3	46.7	
Employment				<0.001
Yes	57.5	48.5	60.7	
No	42.5	51.5	39.3	
Urbanisation				<0.001
Urban	80.5	83.6	79.4	
Rural	19.5	16.4	20.6	
Indigenous				0.011
Yes	1.4	1.9	1.3	
No	98.6	98.1	98.7	
Number of children under 18 years living at the residence				<0.001
None	64.9	80.0	59.6	
One or more children	35.1	20.0	40.4	
Relationship status	100.0	25.9	74.1	
**Healthy lifestyle factors, %**				
Physical activity (meeting recommendations)	58.5	57.5	58.9	0.151
TV viewing (≤14 hours/week)	41.9	39.7	42.7	0.001
Fruit and vegetable (meeting recommendations)	12.4	11.8	12.6	0.249
Fast food (none in the last week)	56.6	56.3	56.7	0.706
Smoking (non-smoker)	85.8	79.6	88.0	<0.001
Alcohol (meeting recommendations)	69.4	64.7	70.8	<0.001
BMI (normal weight)	37.9	42.1	36.6	<0.001

### Frequency of healthy lifestyle factors by relationship status

Frequency of healthy lifestyle factors by relationship status are presented in Table 2. Table 3 presents healthy lifestyle factors by relationship status adjusted for the other healthy lifestyle factors. Physical activity and TV viewing were not associated with relationship status. Alcohol and fast food consumption, fruit and vegetable intake, smoking status and BMI were all significantly associated with relationship status. Compared to singles, couples were significantly more likely to be non-smokers (OR = 1.82, *p* < 0.001), meet the Australian recommendations for fruit and vegetable intake (OR = 1.24, *p* < 0.01), limited fast food (OR = 1.12, *p* < 0.05) and alcohol consumption (OR = 1.27, *p* < 0.001). However, couples were significantly less likely to have a normal weight (OR = 0.81, *p* < 0.001) compared to singles. Fruit and vegetable intake was not significantly associated with relationship status after adjusting for the other healthy lifestyle factors.

**Table 2:** Frequency and trends in healthy lifestyle factors by relationship status.

	Frequency across all years^a^	Trends 2005–2014^b^	
	Couples (Ref: singles) OR (95% CI)	Singles OR (95% CI)	Couples OR (95% CI)
**Physical activity**			
Meeting recommendations	1.08 (0.99–1.17)	0.99 (0.96–1.01)	0.97 (0.95–0.98)***
Not meeting recommendations	1	1	1
**Fruit and vegetable**			
Meeting recommendations	1.24 (1.07–1.43)**	0.98 (0.91–1.06)	0.95 (0.91–0.99)*
Not meeting recommendations	1	1	1
**Fast food**			
None	1.12 (1.01–1.24)*	0.99 (0.94–1.05)	0.96 (0.92–0.99)**
At least once last week	1	1	1
**Smoking**			
None-smoker	1.82 (1.63–2.03)***	1.12 (1.09–1.15)***	1.03 (1.01–1.05)*
Smoker	1	1	1
**Alcohol**			
Meeting recommendations	1.27 (1.12–1.41)***	1.08 (1.04–1.13)***	1.02 (0.99–1.04)
Not meeting recommendations	1	1	1
**TV time**			
≤14 hours/week	1.05 (0.97–1.14)	0.85 (0.83–0.87)***	0.84 (0.83–0.85)***
>14 hours/week	1	1	1
**BMI**			
Normal weight	0.81 (0.75–0.88)***	0.97 (0.95–0.99)*	0.97 (0.95–0.98)***
Overweight/obese	1	1	1

^**a**^Adjusted for sex, age, education, employment, urbanisation, Indigenous status, number of children under 18 years living at the residence and survey year.

^**b**^Adjusted for sex, age, education, employment, urbanization, Indigenous status and number of children under 18 years living at the residence.

*p < 0.05

**p < 0.01

***p < 0.001

**Table 3:** Frequency and trends in healthy lifestyle factors by relationship status adjusted for other healthy lifestyle factors.

	Frequency across all years^a^	Trends 2005–2014^b^	
	Couples (Ref: singles) OR (95% CI)	Singles OR (95% CI)	Couples OR (95% CI)
**Physical activity**			
Meeting recommendations	1.04 (0.92–1.17)	0.98 (0.91–1.06)	1.00 (0.96–1.04)
Not meeting recommendations	1	1	1
**Fruit and vegetable**			
Meeting recommendations	1.16 (0.96–1.40)	0.95 (0.84–1.08)	0.99 (0.93–1.06)
Not meeting recommendations	1	1	1
**Fast food**			
None	1.16 (1.02–1.32)*	1.08 (0.97–1.18)	0.97 (0.93–1.02)
At least once last week	1	1	1
**Smoking**			
Non-smoker	1.71 (1.43–2.04)***	1.10 (1.99–1.21)	1.09 (1.02–1.16)*
Smoker	1	1	1
**Alcohol**			
Meeting recommendations	1.18 (1.02–1.36)*	1.14 (1.05–1.25)**	1.04 (0.99–1.09)
Not meeting recommendations	1	1	1
**TV time**			
≤14 hours/week	0.90 (0.79–1.04)	0.48 (0.44–0.52)***	0.47 (0.45–0.50)***
>14 hours/week	1	1	1
**BMI**			
Normal weight	0.75 (0.67–0.85)***	0.98 (0.91–1.06)	1.03 (0.99–1.08)
Overweight/obese	1	1	1

^**a**^Adjusted for sex, age, education, employment, urbanisation, Indigenous status, number of children under 18 years living at the residence, survey year and all other healthy lifestyle factors.

^**b**^Adjusted for sex, age, education, employment, urbanization, Indigenous status, number of children under 18 years living at the residence and all other healthy lifestyle factors.

*p < 0.05

**p < 0.01

***p < 0.001

### Trends in healthy lifestyle factors by relationship status

**Fig 1:**
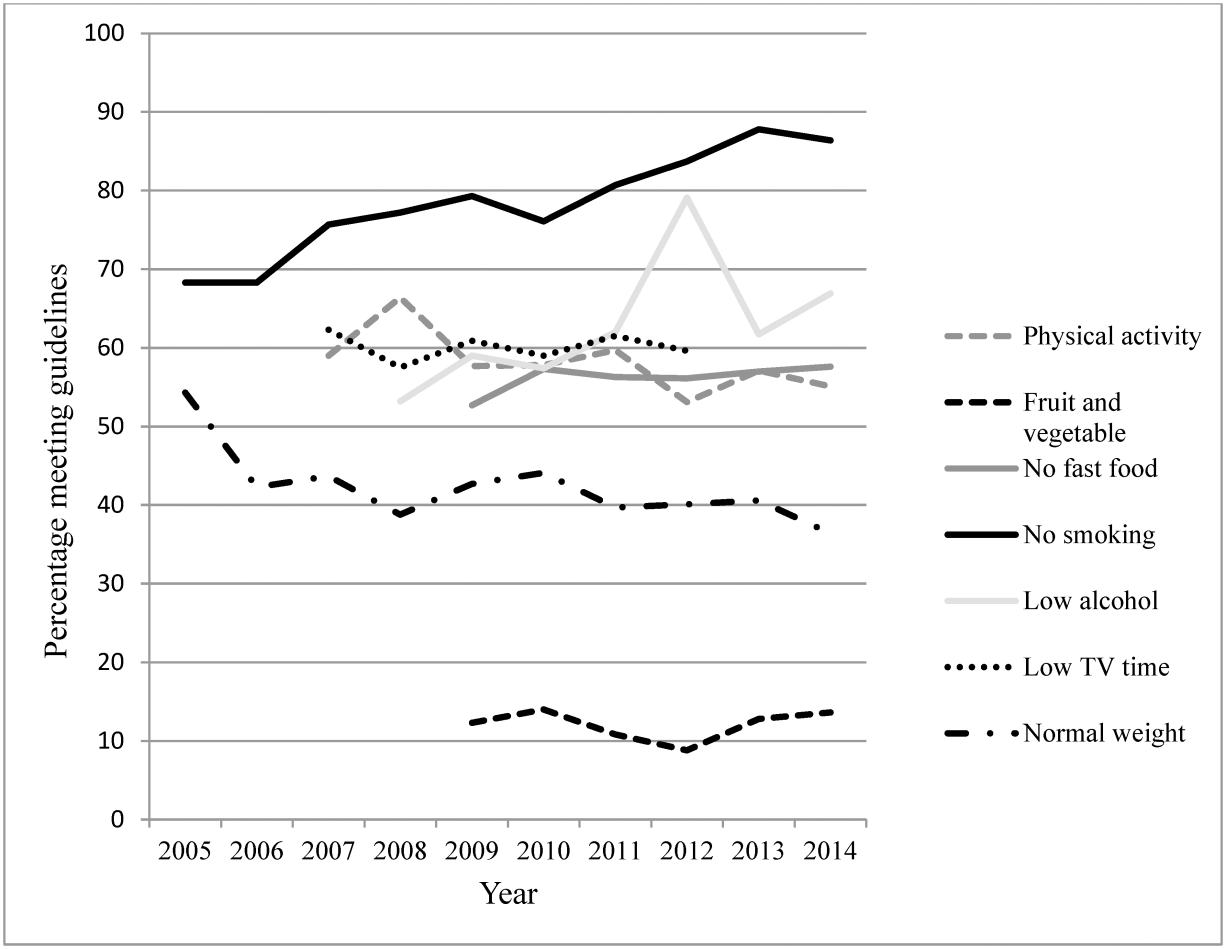
Trends in healthy lifestyle factors in singles.

**Fig 2:**
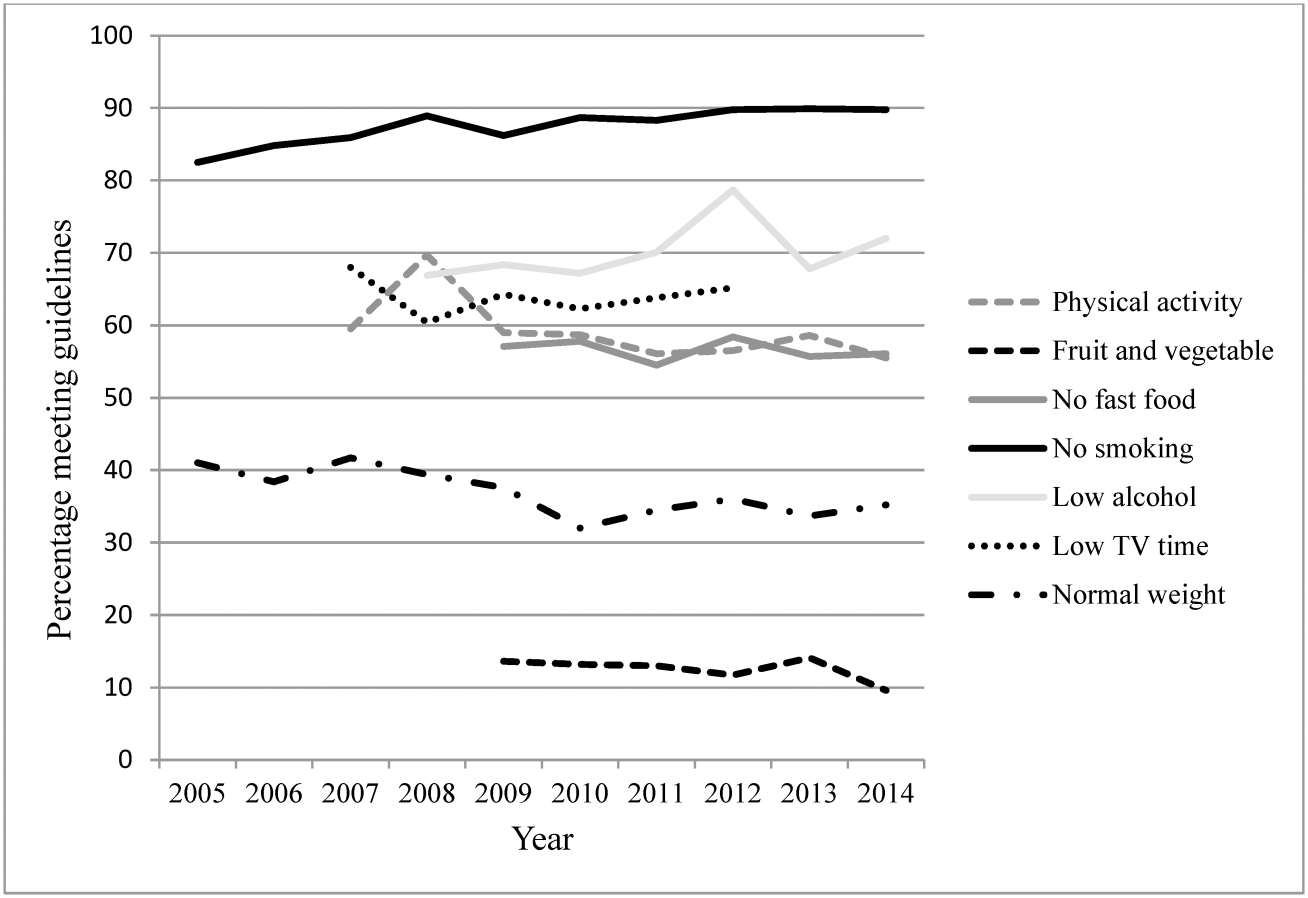
Trends in healthy lifestyle factors in couples.

Trends in healthy lifestyle factors by relationship status are presented in Table 2, and Figs 1 and 2. Table 3 presents trends in healthy lifestyle factors by relationship status adjusted for the other healthy lifestyle factors. Significant trends were observed in relation to all lifestyle factors. Some trends were observed in both couples and singles, whilst other trends were present in either singles or couples. In both singles and couples, rates of normal weight significantly declined (singles: OR = 0.97, *p* < 0.05; couples: OR = 0.97, *p* < 0.001), as did TV viewing time ≤14 hours per week (singles: OR = 0.85, *p* < 0.001; couples: OR = 0.84, *p* < 0.001). The BMI trend was no longer significant when adjusting for health behaviours. Contrastingly, non-smoker rates increased (singles: OR = 1.12, *p* < 0.001; couples: OR = 1.03, *p* < 0.05). This trend was no longer significant in singles when adjusting for the other healthy lifestyle factors. In singles, rates of meeting alcohol recommendations significantly increased (OR = 1.08, *p* < 0.001). In couples, rates of meeting physical activity, and fruit and vegetable recommendations significantly decreased (OR = 0.97, *p* < 0.001 and OR = 0.95, *p* < 0.05, respectively), as did the rates of eating no fast food (OR = 0.96, *p* < 0.01). The physical activity, fruit and vegetable and fast food trends in couples were not significant when adjusting for the other healthy lifestyle factors.

## Discussion

This study investigated the frequency of healthy lifestyle factors in Queensland singles and couples and identified trends in these healthy lifestyle factors between 2005–2014. The frequency data, that was unadjusted for other healthy lifestyle factors, showed that compared to singles, couples were more likely to engage in healthy behaviours (i.e., meeting recommendations for fruit, vegetable, fast food and alcohol intake, being non-smoker). Conversely, couples were less likely to have a normal weight compared to singles. The unadjusted trend data showed that amongst both couples and singles, the percentage who maintained a normal weight and limited their TV viewing to ≤14 hours per week declined, whilst the percentage who were non-smokers declined. Some trends occurred in either singles or couples. For example, in couples, rates of meeting physical activity and fruit/vegetable recommendations significantly decreased, as did the rates of eating no fast food. In singles, rates of meeting alcohol recommendations significantly increased. After adjusting for other healthy lifestyle factors, the higher rates of meeting the fruit and vegetable recommendations in couples was no longer significant. Further, the BMI trends in couples and singles, the smoking trend in singles, and the physical activity, fruit/vegetable and fast food trends in couples were no longer significant.

The frequency data, that was unadjusted for other healthy lifestyle factors, showed that couples were more likely to meet recommendations for fruit, vegetable, fast food and alcohol consumption, and they were more likely to be a non-smoker. These findings are consistent with the results from previous research [10,11,13,29] showing that being in a committed romantic relationship is associated with health promoting behaviours. Previous studies [10,11,29,43] have also observed higher fruit and vegetable intake, and lower alcohol and tobacco consumption among couples. A possible explanation for this consistent finding is that spouses exert influence over each other’s behaviour and provide social support for health promoting behaviours [33,44,45]. In relationships, behaviour patterns are being consciously negotiated; hence, (un)healthy behaviours are brought into focus [30] which may prevent the other from engaging in unhealthy behaviours such as the frequent consumption of high-calorie food, tobacco and alcohol [22]. Furthermore, smoking, excessive drinking and high-calorie food intake are often related to higher levels of stress [46]; social support from a partner may act as a buffer against the harmful effects of stress, thereby reducing the likelihood of engaging in unhealthy lifestyle behaviours [12].

The association between fast food consumption, alcohol consumption, and smoking by relationship status remained when adjusting for the other healthy lifestyle factors. However, no association between fruit and vegetable intake and relationship status was observed in the adjusted analysis. Past research has found a significant association between fruit and vegetable intake and relationship status, however, other healthy lifestyle factors were not adjusted for [10]. The results of the adjusted analysis suggests that the relationship between fruit and vegetable intake and relationship status could be influenced by fast food consumption, alcohol or smoking differences by relationship status.

No differences were observed for physical activity by relationship status in either the unadjusted or adusted analsysis. This is not consistent with previous research which found that physical activity decreased when people entered into relationships [33]. Fast food consumption, drinking alcohol and smoking are negative health behaviours which may be more noticeable by a spouse when compared to insufficient physical activity [29, 45]. Therefore, it is possible that this finding is due to people being more likely to encourage their partners to reduce fast food, alcohol and smoking than encourage them to be more active [33].

The weight status by relationship status analysis, both adjusted and unadjusted for other healthy lifestyle behaviours, demonstrated that couples were less likely to have a healthy normal weight. This finding is consistent with the results from many [22,23,32,47] but not all studies [8,23] examining associations between relationship status and overweight. There are several explanations for this finding. Marriage (or de-facto relationships) comes with spousal obligations such as regular family meals [48]. Dining together relative to dining alone can have positive and negative dietary outcomes. For example, whilst family meals may include more healthy foods such as fruits and vegetables and less fast food [30], people often consume larger portion sizes and more calories in the company of others than they do alone [49], resulting in increased energy intake [32]. Further, the unhealthy but tempting eating habits of one spouse may migrate to the other. For example, Worsley [50] showed that husbands detrimentally influence the diet of their wives by increasing the consumption of fat and meat. It is also possible that the presence of children in the household may expose parents to snack food, increase the consumption of sweets, or may bring parents to eat their children’s leftovers on the plate, all of which may lead to weight gain [32]. Another interesting explanation is the marriage-market theory [22] which suggests that married people who are no longer concerned with attracting a mate gain weight. Entry into cohabitation or marriage is associated with a decline in the desire to maintain weight for the purpose of attracting a mate [20].

The trend data, that was unadjusted for other healthy lifestyle factors, showed that in both singles and couples, rates of normal weight and TV viewing ≤14 hours per week have declined, whilst non-smoking rates have increased. These trends are consistent with those observed in the general Australian population. National data has shown that overweight/obesity and TV time have increased in Australian adults [51–53], whilst smoking rates have decreased [54]. Reasons for these trends are manifold, but some are worth noting. For example, Australia’s progressive tobacco control policies (e.g. tobacco tax, smoke free public places, plain packaging) may have contributed to the decline in smoking [55]. The increased availability of multiple screens (TV, laptops, and tablets) and online TV in most households [56] may have promoted the rise in TV time. The ready availability of cheap, high kilojoule processed foods that are aggressively marketed has likely contributed to the rise in obesity [51]. However the BMI trend in both singles and couples and the smoking trend in singles were not significant when adjusting for other healthy lifestyle factors. Therefore, the increase in overweight and obesity may be partially explained by the decline in health behaviours such as <14 hours TV viewing. Likewise, the increase in non-smoking rates in singles may be influenced by the increase in other health behaviours such as meeting alcohol recommendations.

Whilst the frequency data, both unadjusted and adjusted for other healthy lifestyle factors, showed that singles consume more alcohol than couples, the trend data, both unadjusted and adjusted, revealed that rates of meeting alcohol recommendations have significantly increased over time in singles. This trend is in line with the decline in alcohol consumption in the general Australian population [54]. It may be that many singles are middle-aged or older adults who have entered a period in their life where excessive drinking whilst socialising has become less appealing. As such, the increase in meeting alcohol recommendations amongst singles could partially be driven by reaching a more mature age and transformation from heavier drinking to lighter drinking stages during the life-course [57].

To our knowledge, this is one of few studies [11,22] to have investigated frequency and trends in multiple healthy lifestyle factors, among singles and couples. The findings can improve the targeting of health behaviour interventions in adults. Other methodological strengths of this study include the use of a large sample, validated measures where available, adjustment for potential confounders, and examining multiple healthy lifestyle factors together. Our sample was fairly representative of the general Australian adult population which in 2011 presented with a median age of 43 years, 54% females, 71% couples, 3% Indigenous people, 52% having more than 12 years education, 62% having paid employment and 90% living in urban areas [26,57]. This study also had limitations. First, the cross-sectional design precludes inference on causal relationships and identification of healthy lifestyle changes ‘within individuals’. Second, self-reports of healthy lifestyle factors used in the population surveys are typically prone to social desirability and recall bias. Third, whilst the annual response rates for participation in the Queensland Social Survey (32–44%) were comparable to those reported in many telephone surveys [35], the lower response rates may have introduced non-response bias, i.e. survey responders may have differed from non-responders in terms of sociodemographic and healthy lifestyle factors. Fourth, the scope of this study was limited to the general population of singles and couples without further disaggregation of the data by sex, age and additional information about relationship status (e.g., length of the relationship). Sex and age are strong predictors of health behaviour and weight status [7,8], and as such, singles’ and couples’ healthy lifestyle factors will likely differ by sex and age-group. It is also possible that the longevity of a relationship influences weight status and health behaviours, as for example, The and Gordon-Larsen [20] found that living with a romantic partner for more than two years was associated with concordant obesity, inactivity, and sedentary behaviour in couples. Finally, it should be acknowledged that whilst the findings from our study have high public health relevance, the strength of associations between relationship status and healthy lifestyle factors was mostly weak to modest (albeit significant), except for smoking which showed a strong association with relationship status. Future studies in this area should use objective measures where possible for assessing healthy lifestyle factors in order to reduce the possibility of social desirability and recall bias. In addition, it is worth exploring differences in healthy lifestyle factors among single and couple men and women, as well as those in young, middle-aged and older age groups. If differences exist, public health interventions could target specific population groups, for example single men, and tailored messages could be imbedded in interventions for participants depending on their relationship status.

## Conclusions

This study contributes to the literature by examining the frequency and trends of multiple healthy lifestyle factors together, among singles and couples. Frequency data, that was unadjusted for other healthy lifestyle factors, showed that couples were more likely to pursue healthy behaviours (more fruits and vegetables, less fast food and alcohol, no smoking), but despite this, they were more likely to be overweight, compared to singles. The association between relationship status and fruit and vegetable intake was not significant when adjusting for other healthy lifestyle factors. Unadjusted trend data revealed that in both singles and couples rates of normal weight and TV viewing ≤14 hours per week declined, whilst non-smoker rates increased. However the BMI trend was not significant when adjusting for health behaviours. In couples, unadjusted but not adjusted rates of meeting physical activity and fruit/vegetable recommendations declined, as did rates of eating no fast food. In singles, both unadjusted and adjusted rates of meeting alcohol recommendations increased. Overall, these findings suggest that health behaviour interventions are needed in both singles and couples, but relationship status needs to be considered in interventions targeting alcohol, fast food, smoking and BMI. Research is also needed to understand why health behaviours differ by relationship status in order to further improve interventions.
